# COVID-19 Clinical Research

**DOI:** 10.33696/Signaling.1.006

**Published:** 2020-05-05

**Authors:** Jeffrey Chi, David Chitty, Meeyoung Lee, Nausheen Hakim, Shamsah Lakhani, Lakshmi Rajdev, Xinhua Zhu, Muhammad Wasif Saif

**Affiliations:** Northwell Health Cancer Institute, Donald and Barbara Zucker School of Medicine at Hofstra, Feinstein Institute for Medical Research, Lake Success, NY, USA

**Keywords:** COVID-19, Immunosuppression, SARS-CoV-2, Immunomodulatory, Cytokine storm, Convalescent plasma, Vaccines, BCG

## Abstract

**Introduction:**

While the global COVID-19 pandemic has challenged the entire humanity and health systems, it also triggered researchers to urgently perform clinical trials to assess the safety and efficacy of many agents and modalities to combat COVID-19. As of April 22, over 650 clinical studies have been registered both in USA and internationally. Results from these studies are also coming at a brisk pace in this unprecedented emergency.

**Areas covered:**

We searched the NCI website and Medline and summarize various national and international clinical trials and summarize few of the pivotal ones in this paper, including those specific to oncology population. Two hundred and eighty four studies are actively recruiting adults and children with confirmed COVID-19, including 25 are early-phase I/phase I, 72 phase II, 58 phase III, 12 phase IV, and 31 other trials. They can be categorized into four groups: drugs that combat SARS-CoV-2, immunomodulatory agents to counteract cytokine storm, convalescence plasma therapies and vaccines trials.

**Expert opinion:**

It is hoped that these efforts will results in a successful treatment to COVID-19, especially in a timely fashion before the second pandemic expected in fall. It is essential to acknowledge the devotion and hard work of the clinical research team and clinical research volunteers.

## Introduction

Severe acute respiratory syndrome coronavirus 2 (SARS-CoV-2) causes the novel coronavirus disease 2019 (COVID-19) which was first detected in Wuhan, China in December 2019. Since then it has spread to more than 200 countries. As of April 20, 2020, there have been more than 2.5 million reported cases and 170,000 deaths worldwide [1]. SARS-CoV-2 is a positive sense, single-strand enveloped RNA virus that shares close genetic similarity to corona viruses from previous outbreaks - the severe acute respiratory syndrome corona virus (SARS-COV) and Middle East respiratory syndrome coronavirus (MERS-CoV). The common manifestations from COVID-19 are cough (86.1%), fever or chills (85.0%), and shortness of breath (80.0%) which are very similar to influenza but the case fatality rate of COVID-19 is much high and estimated to be at 3–10%. Diabetes mellitus, cardiovascular disease, hypertension, chronic lung disease, and obesity are some of the prevalent conditions that may increase the risk of hospitalizations [2]. The severe cases of COVID-19 with high mortality are associated with reduced innate and adaptive immune responses in conjunction with abundant cytokine and overexpression of interleukin 6 (IL-6) [3,4]. These patients present with lymphopenia with markedly decreased number of CD4 and CD8 T cells, natural killer cells and monocytes [4]. The United States Food and Drug Administration (FDA) allowed the use of two drugs in treating patients with COVID-19 -chloroquine or hydroxychloroquine via “emergency use authorization” and remdesivir via “compassionate use” in treating patients with COVID 19.

Although there are many ongoing clinical trials, currently there is no strong evidence from randomized clinical trials (RCTs) available. In this editorial, we summarize the ongoing clinical trials, treatment strategies, and clinical experience in treating COVID-19 with a focus on oncology patients.

## The Current Landscape of Clinical Trials for COVID-19

Currently there are 657 registered clinical trials related to SARS-CoV-2 as of April 20th, 2020. Of these trials, 284 are actively recruiting adults and children with confirmed COVID-19 for in-patient and outpatient pharmacological interventions. Of the 284 trials, 25 are Early-phase I/Phase I, 72 Phase II, 58 Phase III, 12 Phase IV, and 31 other trials. They can be categorized into 1) drugs that combat SARS-CoV-2, 2) immunomodulatory agents to counteract cytokine storm, 3) convalescent plasma therapies and 4) vaccines trials (Table 1).

## Trials with Drugs against SARS-CoV-2 Viral Replication

Many agents in this category are already approved by the FDA for treating other diseases. They have generally have proven safety profile and tolerability from previous clinical experience. *In vitro* studies of these agents showed antiviral activities against SARS-VoV-2 and they are now repurposed for treating COVID-19 in clinical trials (Table 1).

Chloroquine and its derivative, Hydroxychloroquine, have been widely used to treat malarial and autoimmune disease. Chloroquine blocks virus infection by raising endosomal pH required for virus-cell fusion and interferes with SARS-CoV-1 receptor glycosylation [5]. It has also been shown to have similar inhibitory effects against SARS-CoV-2 [6]. A recent Chinese study showed that Hydroxychloroquine was more potent and tolerable than chloroquine *in vitro* [7]. In a recent open-label, non-randomized trial, Gautret et al. showed significant reduction in viral load in COVID-19 patients treated with hydroxychloroquine [8]. Another observational study with 1061 patients who received hydroxychloroquine and azithromycin showed good viral clearance and safety profile [9]. Currently, there are 101 clinical trials worldwide using hydroxychloroquine either by itself or in combination with other agents for treating COVID-19 [10].

Remdesivir is an adenosine analogue. It causes premature termination of viral RNA replication when incorporated into growing RNA. It was initially developed as a trial drug for treatment of Ebola during the height of Ebola outbreak. However, the phase 3 randomized trial yielded negative result [11]. Nevertheless, it showed significant *in vitro* antiviral activity against a wide array of RNA viruses in the Flaviviridae and Corona viridae family including SARS-CoV, MERS-CoV and most recently SARS-CoV-2 [6]. There have been reports of anecdotal successful use of remdesivir in COVID-19 patients [12,13]. Currently there are many ongoing clinical trials (e.g. NCT04292899, NCT04292730) to evaluate the efficacy of remdesivir in patients with mild to moderate or severe COVID-19. For severe patients, the data for the first 400 patients in the study was finished, will be released any day (NCT04292899).

Lopinavir-ritonavir is an FDA approved combination antiviral medication used to treat and prevent HIV. It was used in the 2003 SARS outbreak and associated reduced mortality and intubation rates [14]. In terms of COVID-19, early case reports and observational studies showed mix results [14]. However, a recent open label randomized trial in China with 199 patients with COVID-19 failed to show significant difference in viral clearance or 28-day mortality rates [15]. Although there are additional RCTs ongoing, the current data suggest a limited role of the drug in COVID-19 treatment.

Arbidol (Umifenovir) targets S protein/ACE2 interaction, then inhibits membrane fusion of the viral envelope. It is approved for treatment of influenza in China and Russia. There are total of 4 trials available with 2 recruiting for COVID-19 patients.

Camostat mesylate inhibits transmembrane serine protease and prevents viral cell entry. It is primarily used to treat postoperative reflux esophagitis and acute pancreatitis in Japan. There are 4 trials available with 2 recruiting for CoViD-19 patients.

## Immunomodulatory Agents against Cytokine Storm

One of the hallmarks of COVID-19 disease course is that is that the patients can rapidly develop acute respiratory distress syndrome and multiple-organ failure leading to death. Overwhelming cytokine storm is one to the major contributors to the rapid clinical decline. Potent cytokines such as interleukin (IL) −1, IL2, IL-6, TNFa, IFN-α, β were noted to be markedly elevated in these patients. Various immunomodulatory agents are being studied in patients with severe COVID-19 to abate the cytokine storm.

Tocilizumab, an IL-6 receptor antagonist, approved for treatment of cytokine storm due to CAR-T cell therapy, giant cell arteritis and rheumatoid arthritis (RA), has been used to treat severe COVID-19 patients with cytokine storm. A report of 21 COVID-19 patients treated with tocilizumab showed improvements in terms of respiratory function, rapid deffervescence and early hospital discharge [16]. Ongoing clinical trials include NCT04310228, ChiCTR200002976. Sarilumab, another IL-6 receptor antagonist approved for RA, is also being studied in clinical trials (e.g. NCT04288713) in hospitalized patients with severe COVID-19 (Table 1).

Anakinra is an IL-1 receptor antagonist (IL-1ra) approved for RA and neonatal onset multisystem inflammatory disease (NOMID). It can block IL-1 and the downstream inflammatory effects, decreasing/stopping the cytokine storm. It is being studied in clinical trials NCT04339712, NCT04341584. It is also being studied in combination with emapalumab (NCT04324021), a monoclonal antibody against IFN-γ approved for refractory hemophagocytic lymphohistiocytosis (HLH).

Roxolitinib is an inhibitor of Janus Kinase (JAK) 1 and JAK2 approved for the treatment of myelofibrosis, polycythemia vera, and graft-versus-host disease. The JAK pathway is a potential target as IFN-γ exerts its inflammatory effects via the JAK/STAT pathway. It can block the downstream effects of IFN-γ and other cytokines reducing inflammation. Currently there are ongoing clinical trials (NCT04331665, NCT04338958) to study its efficacy in COVID-19 patients.

Selinexor, a nuclear export protein (XPO1) inhibitor approved for treatment of relapsed/refractory multiple myeloma is another potential agent to treat COVID-19 patients. Zhou et al. showed that both SAR-CoV-2 propagation in host cells and inflammatory transcription factors require functional nuclear transport protein XPO1 [17]. Selinexor can potentially stop viral replication and cytokine storm at the same time. Its efficacy in COVID-19 patients is being evaluated in a clinical trial (NCT04349098).

Acalabrutinib is a Bruton’s tyrosine kinase (BTK) inhibitor approved for treatment of chronic lymphocytic leukemia and mantle cell lymphoma. BTK is a key regulator of production of multiple cytokines and chemokines including TNF-α, IL-6, IL-10, and MCP-1 identified as elevated in severe COVID-19 patients. A new study in Spain is underway (NCT04346199) to study acalaburtinib in COVID-19 patients.

## Convalescent plasma

The use of convalescent plasma and passive immunization has been studied in the treatment of respiratory viral illnesses (influenza, SARS-CoV, MERS-CoV) and preliminary data suggests that convalescent plasma may be of benefit in some patients with COVID-19 [18,19]. Specifically, in a Chinese study of 5 critically-ill COVID-19 patients on mechanical ventilation, a one-time administration of convalescent plasma resulted in clinical improvement of all patients, with 3 patients being weaned off mechanical ventilation within 2 weeks of the transfusion and 2 others remaining in stable condition [19].

As of April 19, 2020, there were 5 registered convalescent plasma therapy clinical trials in the United States for the treatment of severe and critically ill COVID-19 patients (Table 1). COVID-19 convalescent plasma can be isolated from the blood of patients who have recovered from SARS-CoV-2 infection and are believed to have developed humoral immunity against the virus. The collected convalescent plasma can then be transfused into ABO compatible severe or critically ill patients with active COVID-19.

## Vaccine Trials

Effective vaccination to a large portion of the world’s population is needed to effectively end the COVID-19 pandemic. Since the World Health Organization declared the COVID-19 outbreak as a pandemic in early March, tremendous effort and funding have been allocated to developing vaccines against SARS-CoV-2. DNA and RNA-based vaccine platforms can ramp up production rapidly as no cell incubation is required. Developing recombinant-subunit vaccines require cell culture or fermentation process which can take longer. Currently multiple development platforms are being utilized [20]. Experts estimate that it can take 12–18 months to develop a successful vaccine ready for the general population. There are currently as many as 70 vaccine candidates in the preclinical stage. Some of the notable vaccine candidate already in clinical trials are listed below (Table 2).

Bacillus Calmete-Guerin (BCG), a live attenuated vaccine originally used against tuberculosis, is being repurposed against SARS-COV-2. There are several ongoing phase III clinical trials internationally (NCT04327206, NCT04348370, NCT04328441) recruiting healthcare workers as participants.

Ad5-nCoV is an adenovirus type-5 vector-based recombinant vaccine engineered to produce SARS-CoV-2 spike protein developed by CanSino Biologics in China. The vaccine completed recruitment for the phase I trial (single center, open label, dose escalation) in China. It has commenced to a double-blind, phase II RCT (NCT04341389) and plan to enroll 500 healthy subjects.

mRNA-1273, a lipid nanoparticle encapsulated mRNA-based vaccine that encodes for pre-fusion stabilized spike protein of SARS-CoV-2. The open label, phase I trial (NCT04283461) is now recruiting candidates across the United States. The vaccine candidate was developed by ModernaTX and supported by NIH.

ChAdOx1 nCoV-19 is an adenovirus vector-based vaccine engineered to produce SARS-CoV-2 Spike protein. It is developed by Oxford University, and is being studied in a Phase I/II single-blinded, RCT (NCT04324606). The study involves 3 arms with 5 interventions. The study plans to recruit 510 healthy volunteers in England.

INO-4800, a DNA vaccine candidate developed by Inovio Pharmaceuticals, is being studied in an open-label phase I trial (NCT04336410) to evaluate the safe, tolerability and immunogenicity of the vaccine. The vaccine is administered intradermally followed by electroporation. It plans to recruit 40 healthy volunteers in the United States.

LV vaccine (LV-SMENP) is made by modifying dendritic cells with lentivirus vector expressing COVID-19 minigene protein from Covid-19 to activate T cells. The phase I/II study (NCT04276896) plans to recruit 100 participants in Shenzhen, China.

## COVID-19 in Cancer Patients

Patients with cancer are particularly vulnerable with increased mortality associated with SARS-CoV-2 infection relative to the general population. Cancer patients are typically immunosuppressed with high healthcare utilization requirements which likely contributes to high transmission rates and poorer outcomes. Both Chinese and Italian data demonstrated that the mortality rate of cancer patients with COVID 19 were 39% and 20% although the sample size was smaller with different demographics [21,22]. Both the American Society of Clinical Oncology (ASCO) and the American Society of Hematology (ASH) have provided institutional policy recommendations to guide the timing of initiation of treatment and alternatives to standard treatment regimens during this time when resources are limited and risk of potentially life-threatening COVID-19 infection is high. In addition, the FDA has provided general guidance as to the management of clinical trials during the pandemic, many of which are no longer accruing or temporarily on hold. At the present time, cancer patients are eligible for the vast majority of COVID-19 clinical trials regardless of cancer directed treatment status. In the United States there are currently 2 intervention studies specifically designed for cancer patients and there are 8 observational studies specifically enrolling cancer patients (Table 3).

## Summary

The COVID-19 pandemic is wreaking havoc across the world, causing high number of morbidities and mortalities. It is the greatest global health crisis of our generation. The case fatality rate is estimated to be 3 – 10%. However, given the extremely high infectious rate, there are now over 2.5 million confirmed cases with staggering 175,000 deaths worldwide as of April 20, 2020. For oncology patients with COVID-19, the case fatality rate is even higher, especially those who are on active chemotherapy treatment. The mortality rate of hospitalized COVID-19 cancer patients can be as high as 30% (our own institutional data; n=30). Heath care centers across the globe are using various agents to treat COVID-19 as there are no effective therapies to date with strong evidence. However, it is encouraging to see the high volume and speed of the ongoing clinical trials. High quality evidence-based guidelines are needed urgently to standardize the treatment of COVID-19 especially for this vulnerable oncology patient population.

## Figures and Tables

**Table 1:** Summary of agents that are under investigation and number of available studies. Total numbers of trials and recruiting and phases may vary depending on the trial designs. https://clinicaltrials.gov

	Sponsors/Developers	Class of Agent	Approved use	Number of active trials (N)	Current Phases
**Drugs against COVID19**					
Hydroxychloroquine (HCQ)/chloroquine (CQ)	Available as generic drug	Antiviral Inhibit viral entry and endocytosis as well as host immunomodulatory effects	Lupus erythematosus, Rheumatoid Arthritis, Malaria	N=45, 21/45 recruiting 22/45 not yet recruiting 3/45 enrolling by invitation 1 completed	PhaseI=2 Phase II=18 Phase III= 22 Phase IV=7 Not applicable=2
Remdesivir (GS 5734)	Gilead Science	Antiviral-Adenosine nucleotide Analogue Block RNA dependent RNA polymerase	Conditionally approved for Ebola in the US	N=10, 7/10 recruiting 1/10 enrolling by invitation 1/10 terminated 1/10 suspended	Phase I=1 Phase II=2 Phase III=8
Kaletra (LOPINAVIR-Ritonavir)	Abbott Laboratories	Antiviral-HIV protease inhibitor	HIV	N=31, 17/30 recruiting 9/30 not yet recruiting 1/30, not recruiting 2/30 enrolling by invitation 1/30 completed	Phase I=1 Phase II=10 Phase III=11 Phase IV=6 Not applicable=5
Umifenovir	Pharm standard	Antiviral	Approved to treat influenza in China and Russia	N=4 2/4 recruiting 1/4 enrolling by invitation ¼ not yet recruiting	Phase I=0 Phase II=0 Phase III=0 Phase IV=3 Not applicable=1
Camostat Mesylate	Ono Pharmaceutical	Serine protease inhibitor	Chronic pancreatitis, postop reflux esophagitis	N=4 2/4 recruiting 2/4 not yet recruiting	Phase I=1 Phase II=1 Phase III=1 Phase IV=1
**Drugs against cytokine storm**					
Actemra (Tocilizumab)	Genentech	Monoclonal antibody - IL-6 inhibitor	Cytokine Release Syndrome, Giant Cell Arteritis, Polyarticular juvenile idiopathic arthritis, Rheumatoid arthritis, Systemic juvenile idiopathic arthritis	N=22 15/22 recruiting 3/22 not yet recruiting 1/22 not recruiting 3/33 enrolling by invitation	Phase I=1 Phase II=13 Phase III=5 Phase IV=1 Not applicable=1
Kineret (Anakinra)	Swedish Orphan Biovitrum (SOBI)	Recombinant/ modified - IL-1 inhibitor	Active Rheumatoid Arthritis, Cryopyrin-Associated Periodic Syndromes	N=6 5/6 recruiting 1/6 not yet recruiting	Phase I=0 Phase II=3 Phase III=3
Kevzara (Sarilumab)	Regeneron	Monoclonal antibody - IL-6 inhibitor	Active Rheumatoid Arthritis	N=8 5/8 recruiting 2/8 not yet recruiting 1/8 not recruiting	Phase I=0 Phase II=6 Phase III=5 (phase II/III trials) Phase IV=1
Selinexor (KPT-330)	Karyopharm Therapeutics Inc	Selective Nuclear Export (SINE) inhibitor	Multiple myeloma, investigation for sarcoma	N=2 1/2 recruiting 1/2 not yet recruiting	Phase II =2
Jakafi (Ruxolitinib)	Novartis	Tyrosine Kinase Inhibitor-JAK1, JAK 2	Myelofibrosis, Polycythemia Vera, Acute graft versus host disease	N=8 4/8 recruiting 4/8 not yet recruiting	Phase I=1 Phase II=4 Phase III=1 Not applicable=1
Calquence (Acalabrutinib)	AstraZeneca	Bruton Tyrosine Kinase Inhibitor	Mantle Cell Lymphoma, Chronic Leukemia, Small Lymphocytic Lymphoma	N=1, not yet recruiting	Phase I=0 Phase II=1 Phase III=0
**Convalescent Plasma Trials**	Various institutions and Blood Centers around the globe			N=84 34/84 recruiting 10/84 not yet recruiting 4/84 not recruiting	Phase I=12 Phase II=35 Phase III=12 Phase IV=1 Not applicable=15

**Table 2:** Summary of trials that are currently in the clinical phases of vaccine development.

Vaccine Trials	Institution/Company	Platform	Trials (N) and status	Phases of Trials
**Bacillus Calmette-Guerin (BCG)** NCT04327206, NCT04348370, NCT04328441	University of Melbourne/ Radboud University/ Massachusetts General Hospital	Live-Attenuated, repurposed vaccine	N=3, 2 are recruiting	Phase II/III
**Ad5-nCov** NCT04341389	CanSino Biologics	Adenovirus vector-based recombinant vaccine	N=2 1/2 recruiting 1/2 not yet recruiting	Phase I=1 Phase II=1
**mRNA-1273** NCT04283461	Kaiser Permanente/ Washington Health Research Institute/ NIH/ ModernaTX	mRNA based vaccine	N=1, recruiting	Phase I
**ChAdOx1 nCoV-19** NCT04324606	University of Oxford	Adenovirus vector-based vaccine	N=2 1/2 recruiting 1/2 not yet recruiting	Phase I/II =1
**INO-4800** NCT04336410	Inovio Pharmaceuticals	DNA vaccine	N=1, recruiting	PhaseI=1
**LV vaccine (LV-SMENP)** NCT04276896	Shenzhen Geno-immune Medical Institute	Lentiviral vector-based vaccine	N=2, recruiting	Phase I = 2 Phase II =1

**Table 3:** Current list of interventional COVID-19 studies specifically enrolling cancer patients in the United States

Study Title	Sponsor	Status	Oncologic Eligibility Criteria	Outcomes
**Anti-Interleukin-8 (Anti-IL-8) for Cancer Patients with COVID-19** NCT04347226	Matthew Dallos	Not yet Recruiting	Documented solid tumor (localized or metastatic) or hematologic malignancy within the last 3 years. Prior definitive treatment for localized disease is allowed (with the exception of non-melanoma skin cancer, low risk prostate cancer or non-muscle invasive bladder cancer) and must have occurred within 3 years of enrollment.	Primary: Time to Improvement in the 7-point ordinal scale Secondary: Time to Death Time to Intubation Proportion of patients requiring ICU admission Mortality at 1 month
**Phase III DAS181 Lower Tract PIV Infection in Immunocompromised Subjects COVID-19 Sub study** NCT03808922	Ansun Biopharma, Inc.	Recruiting	Immunocompromised (history of hematopoietic stem cell transplantation, solid organ transplant, history of treatment for solid or hematologic malignancy or immunodeficiency due to congenital abnormality	Primary: Percent of subjects who Return to Room Air (RTRA) (main study) Percent of subjects with improved COVID-19 Clinical Status Scale (sub-study) Secondary: Multiple (see clinicaltrials.gov)
